# Perceptions of family planning services and its key barriers among adolescents and young people in Eastern Nepal: A qualitative study

**DOI:** 10.1371/journal.pone.0252184

**Published:** 2021-05-26

**Authors:** Navin Bhatt, Bandana Bhatt, Bandana Neupane, Ashmita Karki, Tribhuwan Bhatta, Jeevan Thapa, Lila Bahadur Basnet, Shyam Sundar Budhathoki

**Affiliations:** 1 B.P. Koirala Institute of Health Sciences, Dharan, Nepal; 2 Department of Health Services, Ministry of Health and Population, Kathmandu, Nepal; 3 Nepal Health Sector Support Programme (NHSSP)/DFID/Ministry of Health and Population, Kathmandu, Nepal; 4 Central Department of Public Health, Institute of Medicine, Kathmandu, Nepal; 5 Department of Electronics and Computer Engineering, Institute of Engineering, Tribhuvan University, Lalitpur, Nepal; 6 Department of Community Health Sciences, School of Public Health, Patan Academy of Health Sciences, Lalitpur, Nepal; 7 School of Public Health and Community Medicine, B.P. Koirala Institute of Health Sciences, Dharan, Nepal; 8 Department of Primary Care and Public Health, School of Public Health, Imperial College London, London, United Kingdom; Helen Keller International, SIERRA LEONE

## Abstract

**Introduction:**

Family planning methods are used to promote safer sexual practices, reduce unintended pregnancies and unsafe abortion, and control population. Young people aged 15–24 years belong to a key reproductive age group. However, little is known about their engagement with the family planning services in Nepal. Our study aimed to identify the perceptions of and barriers to the use of family planning among youth in Nepal.

**Methods:**

A qualitative explorative study was done among adolescents and young people aged 15–24 years from the Hattimuda village in eastern Nepal. Six focus group discussions and 25 in-depth interviews were conducted with both male and female participants in the community using a maximum variation sampling method. Data were analyzed using a thematic framework approach.

**Results:**

Many individuals were aware that family planning measures postpone pregnancy. However, some young participants were not fully aware of the available family planning services. Some married couples who preferred ’birth spacing’ received negative judgments from their family members for not starting a family. The perceived barriers to the use of family planning included lack of knowledge about family planning use, fear of side effects of modern family planning methods, lack of access/affordability due to familial and religious beliefs/myths/misconceptions. On an individual level, some couples’ timid nature also negatively influenced the uptake of family planning measures.

**Conclusion:**

Women predominantly take the responsibility for using family planning measures in male-dominated decision-making societies. Moreover, young men feel that the current family planning programs have very little space for men to engage even if they were willing to participate. Communication in the community and in between the couples seem to be influenced by the presence of strong societal and cultural norms and practices. These practices seem to affect family planning related teaching at schools as well. This research shows that both young men and women are keen on getting involved with initiatives and campaigns for supporting local governments in strengthening the family planning programs in Nepal.

## Introduction

An unmet need for family planning results in unintended pregnancies and illegal abortions. This has major health and social implications and is often the leading cause of maternal and child mortality in low-income countries [1, 2]. An estimated 214 million women of reproductive age lack access to contraception resulting in an estimated 67 million unintended pregnancies, 36 million induced abortions, and 76,000 maternal deaths each year [3]. Family planning (FP) is a key intervention to limit these adverse health outcomes [4–6]. Such interventions can prevent 90% of abortions, 32% of maternal deaths, 20% of pregnancy-related morbidity globally, and reduce 44% of maternal mortality in low-income countries [1, 7]. FP reduces adolescent pregnancies, prevents pregnancy-related health risks, and helps to prevent HIV/AIDS [8]. Access to contraception promotes education, raises the economic status of women, and gradually empowers them resulting in improved health outcomes and better quality of life [3, 5, 9, 10].

Global data show that only 32% of married women from low-income countries currently use modern contraceptives [9]. According to the Nepal Demographic Health Survey 2016, the total fertility rate was 2.3 births per woman, which is declining and approaching replacement fertility. This is an important achievement. However, the modern contraceptive prevalence rate (mCPR), which is 43%, is still below the target in Nepal [11]. Nepal has consistently failed to reach the target of mCPR for the past 20 years. The future projection of mCPR for 2030 is 60% [5], which may be a distant dream if the barriers and enablers are not identified on time to strengthen the current efforts.

Expanding the coverage and access to effective contraceptive methods are essential to meet the Sustainable Development Goals and to achieve universal access to reproductive healthcare services by 2030 [11, 12]. For this, the government of Nepal has started a FP program with a focus on increasing the use of FP services and reducing the unmet need [5, 11]. However, various factors negatively influence the delivery of FP services including lack of information, limited awareness of dissemination activities, lack of trained staff, and various cultural and religious factors [13].

Family planning is a choice for many youth, but they often experience barriers such as negative provider attitudes, long distances to healthcare facilities, and inadequate stock of preferred contraceptives [13, 14]. Nepali youth are reluctant to use modern contraceptives due to misconceptions about long-term fertility risks, fear of side effects and overall lack of deeper knowledge [15, 16]. Besides, FP decisions are mostly dependent on male household members, including husbands and other elder members [17, 18]. Married women whose husbands are away as migrant workers face unique contraceptive challenges. When their husbands return home for a few weeks in a year, these women are not prepared with their contraceptives, which can result in unwanted pregnancies [18].

The extrapolation of the available literature on FP use among adults from Nepal and elsewhere suggests that youth is an under-researched population when it comes to FP There is also a dearth of evidence on perception and key barriers to the use of FP measures in this population. Hence, this study aims to identify the perceptions of the FP services and barriers to the use of FP among the youth in Nepal to assist policymakers in designing appropriate interventions to strengthen the family planning programs in Nepal.

## Material and methods

### Ethical considerations

The study received ethical approval from the Institutional Review Committee of B.P. Koirala Institute of Health Sciences, Dharan, Nepal as per the Undergraduate Research Proposal review process (URPRB/01/015). We obtained informed written consent from all participants aged 18 and above. For minors, we obtained assent from the parents of the participants with the participants’ permission. For those who could not read, the information sheet was read aloud by a volunteer, verbal consent was given, and a thumbprint, in the presence of a witness, was used in place of a signature. To maintain the confidentiality of the information and the privacy of the participants, only selected participants and the moderators attended the sessions. Personal identifiers and locator information were not collected, and any identifying information accidentally mentioned was removed from the text before the analysis.

### Study setting

The study was conducted among the participants from Hattimuda village of Morang district in Province One of Nepal. Hattimuda village is a community service area of B.P. Koirala Institute of Health Sciences (BPKIHS), Dharan, Nepal. BPKIHS is a public-funded health sciences university, which follows a teaching district concept adopted as a part of its community-based medical education curriculum. BPKIHS also runs a tertiary hospital service for the population of eastern Nepal [19]. There is a public health facility in Hattimuda village that provides primary health care services including FP services such as the distribution of contraceptives. The nearest secondary and tertiary levels of healthcare services are available 18 kilometers away in Biratnagar, which is the provincial capital and the headquarters of Morang district. According to the 2017/18 annual report of the Department of Health Services, the contraceptive prevalence rate of Morang district is 54.6% [5] whereas the unmet need for FP in Province One as per the Nepal Demographic Health Survey 2016 is 25% [11].

### Study design

This was a qualitative study with an exploratory design to gather a deeper understanding of the perception of FP and its barriers. Focus group discussions (FGD) and in-depth interview (IDI) methods were used. The overall study lasted from November 2017 to October 2018.

### Study population and sampling technique

**Fig 1:**
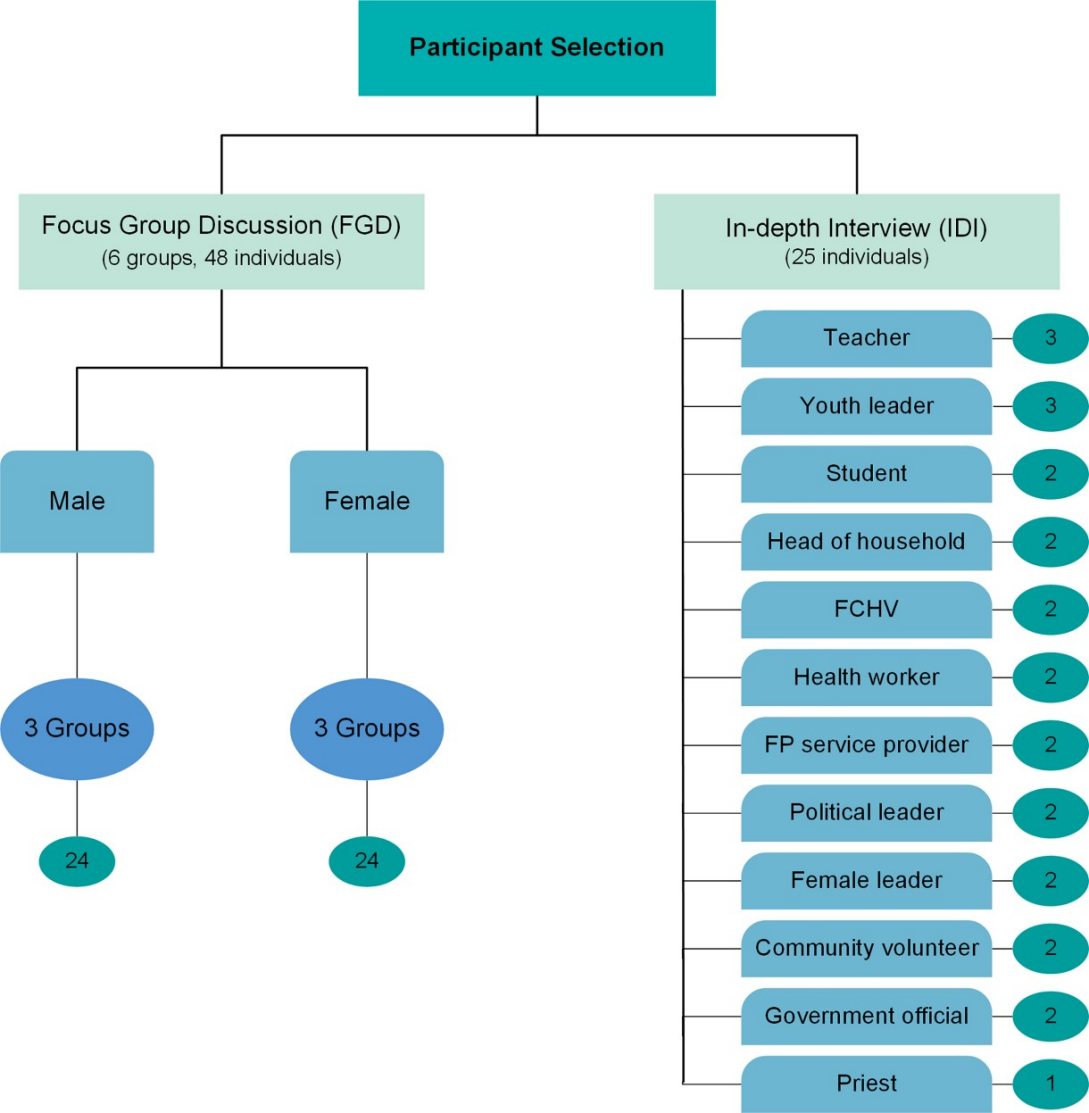
Selection of participants.

Adolescents and young people between 15 and 24 years of age from Hattimuda were included in the study. We used the maximum variation sampling method to enroll participants. Pretesting, including one FGD and four IDIs, was conducted among residents in another village of the same district. The pretesting guided the selection of participants for FGDs and IDIs. Accordingly, FGDs were conducted among adolescents and young people, separately for male and female participants to allow for free expression of views during the discussion of potentially sensitive issues. Moreover, the respondents recommended that people at the forefront of the community such as the village leaders, schoolteachers, community health volunteers, religious leaders, youth leaders, and students be selected for the interviews to gather more information. Along with the recommendations from the pretesting, brainstorming was done with community volunteers to generate a list of people who understood the issues of adolescents and young people. More volunteers were added to the list upon the recommendation of the initial respondents. Thus, participants representing diverse backgrounds in terms of gender, profession, education, and social status, were selected. The IDIs were done among 25 prominent people in the community, which included leaders, school teachers, female community health volunteers, healthcare professionals working at the health post and FP service centers, and youth leaders from youth clubs. Health care providers were included in the interviews as their views would be invaluable due to their experience as FP service providers and as witnessing the health issues faced by youth. The teachers are regarded highly for their knowledge and opinions in Nepali communities. So, they were selected for the IDI to provide more insight into the educational barriers to FP and to help in youth mobilization for FP activities. Considering the vital role of local leaders in influencing the implementation and regulation of population-level activities in the village, they were selected for IDI. Six focus groups were conducted with a total of 48 respondents (Fig 1).

### Data collection

The Focus Group Discussions (FGD) and In-depth interviews (IDI) were conducted by the researchers within the team with prior experience in qualitative research methods. The interview team included an undergraduate medical student, two postgraduate resident doctors, a public health graduate, and a public health academic researcher. Before data collection, an orientation session was conducted for the interviewers using the interview schedule and the topic guide. The IDI guidelines and interview schedules were developed from the literature review and were modified after pretesting. Validation of the tools was ensured by using the Item Objective Congruence (IOC) index and consultation with academics with experience in FP research. Using a semi-structured open-ended questionnaire, the participants were assessed on their knowledge and perceptions regarding sexual and reproductive health (SRH) and FP, SRH problems faced by youth, challenges and barriers to use of FP services, the role of youth in combating the perceived challenges, and suggestions for enhancing the use of services. Data were considered to have reached saturation when the responses from participants became repetitive and/or no new responses were received.

#### Focus group discussions

A representative group of youth from diverse backgrounds who could provide credible information about practices and factors affecting the use of FP in the community was selected. Separate FGDs were held for girls and boys to allow for free expression. A moderator was responsible for guiding the discussion and a note-taker for taking the notes, including recording non-verbal responses and ensuring the audio recording. A total of 6 FGDs, each containing 8 homogenous participants, were conducted. Each individual participated once in the FGD. Every member of the group could make their contribution to any question posed before proceeding to another question. Each FGD lasted for 60–90 minutes on average. The discussion was done in the Nepali language as preferred by participants and later translated into English during transcription.

#### In-depth interviews

In-depth interviews with the key stakeholders were conducted using the Interview Schedule after obtaining the informed consent and audio-recorded with participant permission. A total of 25 IDIs were conducted for the average duration of 30–45 minutes, at a location convenient to the participant, which included their homes and offices.

### Data management and analysis

A framework method of thematic analysis was used. The analysis included stages of transcription, familiarization with the interview, coding, developing a working analytical framework, applying the analytical framework, charting the data into the framework matrix, and interpretation of the data. The data collected from the focus groups and interviews were transcribed verbatim. The notes taken were used as a guide to segregate the responses by different respondents during the discussion. An independent researcher conversant in the Nepali and English languages cross-checked the transcripts for accuracy and preservation of original meaning during translation. Preliminary codes were assigned to the available data and then organized into thematic units that were continually revisited and revised as necessary. To ensure consistency of data and findings, two authors were involved in data analysis and reporting. The recordings were stored and accessed by the research team only and were destroyed after the analysis and final report preparation.

### Operational definition

#### Youth

According to UNFPA, all persons within the age of 15–24 years are considered youth [20].

## Results

The baseline characteristics of the participants can be seen in Table 1.

**Table 1:** Sociodemographic characteristics of the participants.

Variable		FGD		IDI	
		Number	Percentage (%)	Number	Percentage (%)
Age range	15–19 years	23	48	3	12
	20–24 years	25	52	4	16
	Above 24	-	-	18	72
Sex	Female	24	50	12	48
	Male	24	50	13	52
Marital Status	Single	23	47.92	10	40
	Married	25	52.08	15	60
Religion	Hindu	32	66.67	15	60
	Muslim	10	20.83	5	20
	Others	6	12.5	5	20
Ethnicity	Brahmin	14	29.17	8	32
	Chhetri	12	25	6	24
	Indigenous	12	25	8	32
	Others	10	20.83	3	12
Highest level of education	Illiterate	12	25	2	8
	Primary School	15	31.25	5	20
	Secondary School	12	25	10	40
	Bachelor and above	9	18.75	8	32
Profession	Student	12	25	2	8
	Unemployed	20	41.67	6	24
	Employed	16	33.33	17	68
Economic status	Below poverty line	18	37.5	3	12
	Above poverty line	30	62.5	22	88

The responses from the IDIs and FGDs revealed four broad themes. Within each broad theme were several substantive sub-themes that emerged from the data. The themes and subthemes are summarized in Table 2 below.

**Table 2:** Perceptions of FP services and its key barriers among youth.

Themes	Subthemes
**1. Knowledge and perceptions of FP**	a) Sources of information regarding FP • Health workers • Peers • Books • Media (radio, television) b) Perceptions of FP • Inadequate knowledge • Men perceive FP as women’s business • FP for men means the use of condoms
**2. Preference for FP methods and decision-making**	a) Preference among participants • Preference for a traditional method • Methods available in nearby centers b) Decision-making among married participants • Men reluctant to use FP methods • Men are sole decision-makers • Women also expect men not to use FP methods • Some women feel they are physically weak c) Decision-making among unmarried participants • Discuss and joint decision • Requesting husbands use FP methods is disrespectful • Permanent sterilization is simpler for men to adopt
**3. Barriers and challenges in the use of FP**	a) Supply-side barriers and challenges • Inaccessible • Unaffordable • Distant health facilities • Unavailability (stock out) • Lack of youth-friendly FP services • No priority programs from the government • Restrictions on women for participating in FP programs • Outdated school curriculum covering FP • Lack of confidence in teachers to teach FP lessons b) Demand-side barriers and challenges • Lack of awareness • Fear of side effects • Lack of alternative methods other than condoms for men • Lack of easy methods for women • Religious belief, stigma, social pressure • Perceived roles of men and women • Shyness
**4. Role of youth and suggestions to improve FP**	a) Youth’s engagement in strengthening FP services • Engaging interested youth in FP programs • Peer to peer education approach • Training programs • Role-plays/dramas • Counseling sessions • Curriculum update • Mobile outreach clinics • Spousal communication • Gender inequalities • Change in attitude of people b) Suggestions to improve FP services • Establishment of youth centers/clubs • Engaging with male counterparts • Involve key stakeholders from the community • Support from government programs • New methods for men and women with no side effects and high compliance

### Theme 1: Knowledge and perceptions of FP

#### a) Knowledge and sources of information on FP

Participants demonstrated awareness of some form of FP. However, some knew nothing about it. Health workers were commonly referred to as the sources of information, while some also mentioned peers, radio, television, and books. Male participants openly disclosed their sources of information on FP while some female participants were reluctant to share their sources.

#### b) Perceptions of FP

Perceptions of FP varied among participants. Some male participants inferred FP measures as women’s business and did not show any interest in talking more about it. Some referred to FP as using condoms during intercourse, while others referred to oral pills and injectable hormones as FP. Some female participants looked at FP as a way of avoiding unwanted pregnancies.

*“My sister used to say that she has been using injection* (Depo-Provera) *to control unwanted pregnancy*. *I think FP is about the same*.*”-* ***19 years Female*, *FGD participant***

### Theme 2: Preference for FP methods and decision-making

Some female participants reported preference for traditional methods of contraception such as coitus interruptus and calendar method over modern methods. These people used modern methods of FP to start with, which they discontinued later due to the side effects. Participants also stated that the health facilities that provide FP services were far, and hence they had no alternative other than natural methods. Male participants hardly mentioned visiting any health facilities for FP purposes.

*“Most of our clients who come for it (FP) are women. Even condoms are collected by women. Men rarely come alone or as couples for FP services.” -**35 years old Female, FP service provider, IDI participant***

Yet husbands were responsible for the decision-making about FP and choices of methods for most couples. Some participants (both male and females) mentioned that women rather than men should use permanent FP measures. They believed that men being the breadwinner of the family, should not undergo sterilization, for example, as it would make them physically weak.

*“Though I love my wife and I am concerned about her. But I have no options. I must work in a factory. I need to lift heavy weights there. All the major house chores are also done by me. These things (sterilization) would make me weak. How can I earn my livelihood then?”- **22 years Male, FGD participant***

Some female participants expressed their concerns regarding the use of permanent FP methods. They mentioned that they had already been through various phases of pain, be it during menstruation, pregnancy, or delivery which has made them weak. Thus, they prefer their husbands to undertake any measures.

In contrast, unmarried participants stated that they would rather discuss and decide together with their partners regarding which method to choose in the future. Despite this interest, women were not sure how to engage their husbands in discussion. Some female participants said that they could not persuade their future husbands to use contraceptives as it would be disrespectful, whereas a few male participants believed it was a woman’s responsibility to use FP methods.

*“It* (FP) *is stuff to be done by the women*. *So*, *there is no doubt about who would be doing it*. *Moreover*, *people would laugh at me if I do it* ***-20 years Male*, *FGD participant***

“*Women have already gone through much pain in bringing up and taking care of the children and again keeping this stuff (FP) in their head is unjustifiable*. *As such, in comparison to the female operative procedure, I have heard that the male one is simple, less time consuming, and does not bring many complications*. *So, why not we men take the lead on this?*” ***-25 years Male, Youth leader, IDI participant***

### Theme 3: Barriers and challenges in the use of FP

#### a) Supply-side barriers and challenges

Participants indicated that contraceptive services are not always accessible nor affordable in rural areas. Health facilities are far, and many people feel reluctant to travel in a hot climate. Participants who were reluctant to travel said they were doubtful that the health facilities would have the methods in stock even if they managed to walk the distance. Others who were reluctant said they would be unable to afford the contraceptives from a private medical store regularly. A few participants raised the issue of privacy and unavailability of all services at the health centers. Similarly, young males from the community complained that the services at the health post were focused only on mothers and married couples, while the boys and the unmarried people were not given much attention. For this, they suggested changing the term to something other than FP because they believed that FP should include not only those who had families.

Participants expressed their frustration that FP and SRH services in their village had not been running well for more than a year. They felt that the government was not doing anything about it either. Some students expressed the need for an integrated curriculum at school covering every aspect of SRH and FP that would ensure adequate and proper knowledge of such crucial subjects. Despite the students’ desire to learn and understand FP, their teachers are often reluctant to talk about FP in detail. The participants also indicated that family members, in general, forbid girls and women from getting involved in FP awareness activities.

*“Though we are eager to learn about those lessons (reproductive organs and health), our teacher skips them. They tell us to read it by ourselves.”**-18 years Female, FGD participant***

#### b) Demand-side barriers and challenges

A few participants were confused about which method to choose, how to use it properly and did not even know where to seek FP services locally.

*“My husband works abroad. Last year, when he came home during Dashain (festival), we had (intercourse). Later, he returned to his workplace. Meanwhile, I came to know that I was pregnant, after 3 months. I was shocked to hear that. We already had 3 children; 2 of them were unplanned. I did not have enough information about contraceptive measures in this situation. Had I known about them; I would have used them. I had serious trouble travelling to get it aborted.” **- 24 years Female, FGD participant***

Some female participants expressed their reluctance to use FP methods due to their own or other people’s past experiences and the fear of side effects, including vaginal bleeding, spotting, abdominal pain, nausea, vomiting, headache, acne, and infertility. These female participants expressed the need for a single-use FP method with fewer side effects for women which could be used without their husbands’ consent. The male participants were worried about the risk of unwanted pregnancy due to the breaking of condoms and a few participants also expressed concern that they experienced allergic reactions after the use of condoms. Moreover, they were concerned about not having any alternative methods of contraception other than condoms.

*“I have a much bitter experience. I was using Depo injection before. But I started having over bleeding for which I was admitted to the hospital for a few days. Later, I was switched to implants but they also did not suit me. In between I also used pills, but they aggravated my acne and I was feeling nauseated every day. Uff…. I am fed up now. I swear, I won’t ever use any methods.” - **19 years Female, FGD participant***

*“I have heard that keeping these things (Copper-T) in the uterus can cause cancer. Better to avoid it.” **- 20 years Female, FGD participant***

*“There aren’t many choices for men. I think using a condom during sex is like tying plastic around the tongue and eating food.” **- 21 years Male, IDI participant***

Religious and ethnic variation affected use of FP. Participants reported that people belonging to upper caste groups used FP measures more than lower caste groups. Likewise, people who had migrated from the hilly areas used FP services, whereas people from the local ethnic community did not use as they were less aware of it. FP decisions among young people seem to be influenced largely by religious beliefs, stigma, and the perceived role of men and women based on existing social norms. Some participants regarded children as a gift from God and denied using any FP methods. Some believed using FP was going against the law of nature, religion, and culture; thus, they would not avoid childbirth, but rather celebrate every birth. Some indicated that if couples did not have children within 1–2 years of marriage, then people would question the woman’s fertility. Most couples preferred sons to daughters as they believed sons would look after them and their property, while the daughters would be married and sent away, resulting in avoidance of FP measures until they have a son. Some couples even wished to have two sons because if anything unfortunate happened to one, the other son would still be with them to carry the generation forward.

*“My aunt gave birth to a son after 5 successive daughters. She is pregnant again this time in the hope to have a son. She says that she cannot trust to have only one son because if anything happens to their only son, then she will have no one to pay tribute after her death.”-**22 years Female, FGD participant***

Participants also said that people felt shy talking about FP openly. Female participants also felt uncomfortable asking for contraceptives with male health personnel at the health post. Similarly, teachers felt uncomfortable teaching about reproductive health and FP as their children and relatives could be present as students in the classroom. Participants indicated that some students would laugh and smile, making it difficult for the teachers to run the classroom sessions smoothly.

It was reported by a FP service provider that some men opposed their wives using any FP measures as they perceived that the use of FP measures allowed their wives to become promiscuous when they go abroad for work.

*“Some husbands working abroad forbid their wives from using any FP measures because they fear the use of FP measures may provoke a sexual relationship with someone else in their absence”-**30 years Female, Health professional providing medical abortion services, IDI participant***

### Theme 4: Role of youth and suggestions to improve FP

The youth were interested in getting involved in a “peer to peer education” approach to increase awareness among the community about FP use. This approach would include peer training programs, role-plays/dramas, and counseling sessions to break the key barriers linked with such services. Activities ranging from redesigning the school’s curriculum to strengthening FP services in primary care centers, and from launching mobile outreach clinics to facilitating “spousal communication” were intended to change attitudes and support gender equality in sexual and reproductive health. Participants emphasized forming youth centers and collaborating with other youth clubs in the village. Furthermore, they suggested bringing religious leaders, teachers, doctors, and politicians as advisors of the youth centers would be beneficial as they are influential members of the community.

*“I feel bad for my sister who is not given much importance from my parents. She got married against her choice due to her parents’ pressure. Now, they are forcing her to have kids. She is just 15 and if she gets pregnant, what will happen to her health and her child, how can she take care of a baby? I had a long debate with my father yesterday. I have now decided to start a youth club to promote awareness regarding FP and preventing early marriage and teenage pregnancies.” **- 23 years Male, FGD participant***

Male participants indicated that family planning programs are effective only when men prioritize women’s autonomy. Moreover, they expressed disappointment with the local government for not encouraging the involvement of men in FP programs in their village. To help address this issue, they expressed their interest in supporting the local government in bringing inclusive FP programs to their village.

*“For a long time, women have been using those (Contraceptives) by hiding. We are always in fear about what others would say if they came to know about us using it. This can be addressed through male involvement and support.” **-24 years Female, FGD participant***

## Discussion

This qualitative study provides in-depth information on the understanding and perceptions of youth in Eastern Nepal regarding FP. This study generated findings regarding knowledge and perceptions of rural residents regarding FP and its methods; decision-making and preference among participants; supply-side and demand-side barriers and challenges regarding the use of FP measures; steps that can be taken to improve their use; and the role of youth in increasing FP coverage. Although most participants knew something about FP, a few female participants were completely unaware of it. And while some participants agreed that all married couples should be using FP measures, some unmarried male participants believed that those measures should be exclusively for women. These men said that they would let their wives use them after getting married. Current FP methods for men are either coitus-dependent, such as condoms or withdrawal, or permanent, such as vasectomy. Limited choices for men may have resulted in misconceptions that contraceptives are mostly for women.

Men often claimed to be the sole decision-maker of the family on important matters, including those related to family health and contraception. In most circumstances, men solely decide the FP measure to be used without having a discussion with their partner. This might be one of the reasons why women are bound to adopt a FP method that is not necessarily their choice. Besides, this problem is further reinforced by the limited options of FP methods available for men other than condoms and permanent sterilization. These findings are supported by other studies in South Asia, where family planning measures are mostly considered women’s responsibility [21–24]. Health workers, peers, and mass media were the most common sources of information regarding FP similar to prior studies in India [21, 24] and Nepal [22]. Participants in this study seemed to assign FP responsibility to the other gender in terms of using FP. This could mean that there is a gap in communication within the couples when deciding about FP. There is a need for further research to identify ways to improve communication among couples.

Religious and ethnic variation influence FP use. People belonging to privileged ethnic groups used FP measures more than underprivileged groups. This is despite family planning services being free for all citizens in Nepal. In this study, people who had migrated from hilly regions knew about and used FP services more than those belonging to the ethnic community in the local region. This is an area for further research to understand differences in knowledge and perceptions regarding FP between the population groups. This can be argued as a limitation of the current FP promotion programs, which may not have considered the different needs of people from different religious and ethnic backgrounds [25]. A few participants reported that their holy scriptures forbade them from using FP methods as they viewed children as a gift from God; any artificial process interrupting pregnancy or preventing the possibility of life is a religious offense for them [26]. Previous studies from Nepal have shown that this belief has long been rooted in some communities [27–29].

Apart from religious beliefs, fear of side effects, having experienced adverse health consequences after using hormonal contraceptives, and fear of potential infertility in the future are reasons for reluctance using FP methods among women [30]. Besides, we can speculate that language and cultural barriers, and fear of discrimination especially by male counterparts negatively influence the use of FP measures among some women despite their strong interest in using them. The use of IEC materials in raising awareness and empowering married couples for shared decision-making could help generate demand [28, 29]. Local cultural taboos restrict open communication about safer sex measures and sexual health in Nepal, prohibiting young girls and boys from receiving adequate information and guidance regarding sexual and reproductive health and FP [31].

Most of the married women and men stated that the decision-makers of the family are men. The husband decides whether or not to use contraception, or more specifically, whether or not to let their wives use it. However, unmarried participants expressed their willingness to decide mutually with their spouse regarding FP use in the future [21, 32]. Most women in this study seemed comfortable letting their male partners decide on contraceptives. This attitude could be explained by the patriarchal dominance in decision-making [19, 33, 34].

Some men mentioned that condoms inhibit their sexual pleasure, which is why they prefer women to use other methods instead. A study conducted in Far West Nepal and another nationwide study reported similar concerns among men [31, 35]. Adolescent girls stated that they were not comfortable talking to a male health worker about FP or to a female worker in the presence of a male health worker, which has also been reported elsewhere [36]. Some women said that their husbands forbade the use of contraceptives because they thought that contraceptives would allow their wives to become promiscuous and that using FP was a sign of infidelity. This issue, however, was not raised by any men in the study. Some women reported violence as a consequence of using contraceptives without their husband’s consent. Prior qualitative studies also reported that women may suffer domestic violence for opposing their husbands. Studies suggest that a multi-sectoral action involving stakeholders from health, women’s rights, and education sectors is imperative to further research and address this issue [29, 36, 37].

Supply constraints (distance to a provider for getting contraceptives, out of stock, limited choices of contraceptives, unaffordable methods, etc.) could aggravate the unmet need for contraception. These constraints are similar to all regular supplies faced by the health system in Nepal. However, supply-side interventions such as increasing the number of health facilities distributing FP services, policy focusing on consistent operating hours, and full stock of a wide variety of FP methods could largely improve uptake and increase contraceptive coverage [18, 38].

Most female participants did not speak up when asked about their perception of the role of men in FP. On the other hand, male participants explained that the role of the youth could be disseminating FP information, conducting awareness campaigns, organizing dramas and role-plays to educate people about the religious and cultural barriers of FP use, etc. With appropriate training, the young men said they would be willing to work for FP advocacy in the community.

Reproductive health leaders and planners should identify men who are willing to share decision-making authority with their wives and devise behavioral change interventions [39]. Male participation could support the FP programs and also help empower women [40]. The participants in the study expressed the need for the current FP programs to consider the community members as key stakeholders in planning FP programs. There is a need to further explore possible ways of working with the rural, marginalized communities and hard-to-reach or specific ethnic groups to improve their update of FP services [41]. There is evidence that mass media messages increase the likelihood of FP use, which could be considered by advocacy and dissemination programs [42]. Evidence from maternal and newborn health care research shows that interventions that engage men result in more equitable couple communication and shared decision-making. This may be a relatable concept to be considered for FP programs as well [43].

We urge those in charge of the health and sexual education curriculum to find ways to encourage teachers to give equal attention to these topics, including FP education, as they would to any other. It was reported that teachers were reluctant to teach about FP as they perceived the young students felt discomfort around this topic. Further research to identify innovative youth-friendly methods to teach sexual and reproductive health topics to students may be helpful. Youth groups should be regarded as important stakeholders in the redesign of school health curricula, particularly for their insight into culturally sensitive and otherwise effective ways for delivery. Health professionals, members of local organizations, and community leaders pointed to the necessity of addressing unmet FP needs and the stigma associated with FP use through community education approaches that take into account cultural norms and beliefs [44]. Interventions focusing on reproductive health education curricula involving school teachers could be considered [45]. Strengthening health systems, bridging service gaps, improving the integration of contraceptive services and counseling with routine health care are important strategies for increasing contraceptive uptake in eastern Nepal [22].

Among the study’s limitations was the fact that it was conducted in a single village in eastern Nepal. Our findings might differ if the sample had been drawn from other parts of the country. Although participants spoke fluent Nepali, some phrases used in local dialects could not be perfectly translated into Nepali or English. These responses could have been affected by social desirability as the participants may have felt constrained from speaking freely with people from health institutions. To help reduce these obstacles we held open meetings and drop-in sessions with the support of community youth to disseminate the purpose of the study and build rapport with the young people in the village before we approached them for the study. Moreover, participants were assured anonymity and confidentiality, which may have increased their willingness to participate in the research.

## Conclusions

There appear to be information and communication gaps between women and men regarding FP services and programs. The information gap could be addressed by exploring ways to increase information uptake in schools through redesigning the curriculum delivery. Mass media may be used to disseminate appropriate health education regarding FP. Health institutions could consider approaches to create FP information and service centers that are male-friendly. The communication gap may be more deeply rooted in the culture and traditions of Nepalese society. In a mostly patriarchal society, further identification of motivations for men to participate in FP related activities could be challenging. However, it is promising that men may be willing to support their partners for FP decision-making and engage in strengthening FP programs through the “peer to peer” approach via youth-led centers and community clubs. Program managers and policy makers need to take into account the fact that youth are willing to contribute to ongoing FP programs. Doing so would help bridge the information and communication gaps between school education and practice. Innovative research to further explore perceived benefits by youth on the uptake of family planning, sexual and reproductive health services is needed.

## Supporting information

S1 File (DOCX)

S2 File (DOCX)

## Abbreviations

BPKIHS: B. P. Koirala Institute of Health Sciences
FP: Family Planning
FGD: Focus Group Discussion
IDI: In-Depth Interview
mCPR: Modern Contraceptive Prevalence Rate
SRH: Sexual and Reproductive Health
