# Supplementary material for: Perceptions of family planning services and its key barriers among adolescents and young people in Eastern Nepal: A qualitative study
Source: PLoS One. 2021 May 26;16(5):e0252184. doi: 10.1371/journal.pone.0252184 (PMC8153486; doi:10.1371/journal.pone.0252184)
Supplement: S1 File — (DOCX) [file pone.0252184.s001.docx]

# Perceptions of family planning services and its key barriers among adolescents and young people in Eastern Nepal: A qualitative study

# INTERVIEW GUIDE

**Interview Guide for Focus Group Discussion**

1. Concept of family planning
2. Primary source of family planning information
3. Societal perception on family planning
4. Religious or social stigma related to family planning
5. Reasons for female sterilization preference among men
6. Perceived side effects experienced by women after sterilization
7. Decision making about family planning in the family
8. Informative and awareness programs about family planning by the Government/Family Planning Association of Nepal/ District (Public) Health Office/ Non-governmental organizations
9. Role of male/female youths in strengthening the FP services in your community
10. Adequacy of family planning education in high school curriculum
11. Youth led social activities in the community about family planning

**Interview Guide for Focus Group Discussion in Nepali**

1. परिवार नियोजनबारे तपाईँको अवधारणा
2. परिवार नियोजनबारे जानकारीको मूख्य स्रोत
3. परिवार नियोजनबारे समाजको धारणा
4. परिवार नियोजनबारे व्याप्त धार्मिक तथा सामाजिक अन्धविश्वास
5. पुरुष भन्दा महिला बन्ध्याकरणलाई प्राथमिकता दिनुका कारणहरू
6. बन्ध्याकरण गरिसकेपछि महिलामा हुने असरहरू बारे बुझाइ
7. परिवार नियोजन बारेमा तपाईँको परिवारको निर्णायक मान्छे
8. परिवार नियोजन सम्बन्धी नेपाल सरकार/नेपाल परिवार नियोजन संघ/जिल्ला (जन)स्वास्थ्य कार्यालय/गैरसरकारी संस्थाहरूले गर्ने जनचेतनामूलक एवं जानकारीमूलक कार्यक्रम
9. समुदायमा परिवार नियोजनका सुविधाहरूको सुदृढीकरणमा युवाहरूको भूमिका
10. उच्च माध्यमिक विद्यालयको पाठ्यक्रममा परिवार नियोजनबारेको शिक्षा
11. परिवार नियोजनबारे युवाहरूले समुदायमा गरेका सामाजिक कार्यहरू

**Interview Guide for In-depth Interview (IDI)**

1. Perception about family planning?
2. Can you elaborate on the benefits of family planning at individual level?
3. Can you elaborate on the benefits of family planning at community level?
4. Can you elaborate on the benefits of family planning at national level?
5. Are you aware about health effects of not using family planning methods?
6. What do you think are your motivations to use family planning?
7. Please tell about the motivation for regular education?
8. How do you find the willingness of participants to learn about family planning and its methods?
9. Please tell me something about the types of contraceptive devices?
10. Could you elaborate about the stakeholders of a successful family planning program?
11. Where do you see the role of youth in steering the family planning program at community level?
12. Could you explain about your interest in contributing towards family planning?
13. How do you see the role of male in family planning?
14. How do you see the role of female in family planning?
15. Would you say that there is a huge contribution pending due to less male/female participation? Please explain.
16. Tell something about your participation in youth activities for family planning? If yes, explain.
17. What is the source of knowledge for the youth about Sexual and Reproductive Health and family planning? Is it available in the school or campus curriculum?

**Interview Guide for In-depth Interview (IDI) in Nepali**

1. तपाईँलाई परिवार नियोजन बारे के थाहा छ?
2. व्यक्तिगत तवरमा परिवार नियोजनका फाइदाहरू के के हुन्?
3. सामुदायिक स्तरमा परिवार नियोजनका फाइदाहरू के के हुन्?
4. राष्ट्रिय स्तरमा परिवार नियोजनका फाइदाहरू के के हुन्?
5. परिवार नियोजनको अप्रयोगले हुने स्वास्थ्य असरहरू बारे के तपाईँ जानकार हुनुहुन्छ?
6. परिवार नियोजनको प्रयोग गर्न तपाईँ केबाट प्रोत्साहित हुनुभयो?
7. नियमित शिक्षाका लागि तपाईँको प्रेरणाको स्रोत के हो?
8. सहभागीहरूको परिवार नियोजनका बारे जान्न खोज्ने इच्छालाई तपाईँले कसरी नियाल्नुहुन्छ?
9. के तपाईँलाई गर्भनिरोधक साधनहरूबारे ज्ञान छ?
10. सफल परिवार नियोजनका सरोकारवाला को हुन्?
11. परिवार नियोजनलाई सामुदायिक स्तरमा डोर्‍याउन युवाहरूको भूमिका के हुनुपर्छ जस्तो लाग्छ?
12. तपाईँलाई परिवार नियोजनमा योगदान गर्न मन लागेको कुरा विस्तृतमा भन्न सक्नुहुन्छ?
13. परिवार नियोजनमा पुरुषको भूमिकालाई कसरी नियाल्नुहुन्छ?
14. परिवार नियोजनमा महिलाको भूमिकालाई कसरी नियाल्नुहुन्छ?
15. महिला वा पुरुषको न्यून सहभागिताले गर्दा धेरै योगदानहरू रोकिएको बारे तपाईँ केही भन्न चाहनुहुन्छ?
16. के तपाईँले कहिल्यै परिवार नियोजनसँग सम्बन्धित कार्यक्रमहरूमा सहभागिता जनाउनुभएको छ? यदि छ भने, विस्तृतमा भन्नुहोला ।
17. परिवार नियोजन तथा यौन शिक्षाको बारेमा युवाहरूका लागि सूचनाको स्रोत के हो? के यस सम्बन्धी विषयवस्तु स्कुल तथा क्याम्पसको पाठ्यक्रममा समावेश गरिएको छ?
