# Supplementary material for: Perceptions of family planning services and its key barriers among adolescents and young people in Eastern Nepal: A qualitative study
Source: PLoS One. 2021 May 26;16(5):e0252184. doi: 10.1371/journal.pone.0252184 (PMC8153486; doi:10.1371/journal.pone.0252184)
Supplement: S2 File — (DOCX) [file pone.0252184.s002.docx]

| Perceptions of Family Planning and its key Barriers | |
| --- | --- |
| **Knowledge and perceptions regarding family planning** | |
| Knowledge and sources of information | - *F1: The community sister (FCHV) had told me. She time and often comes to my home to look after my sister-in-law who is pregnant. At the time she was explaining her, I also listened to her.* - *F10: Moreover, I knew from my wife. She was told by the nurse in the hospital where she went last time to get herself checked for her first baby. Then she shared with me.* - *F8: Before marriage… I had just heard about it* (family planning) *in some female talks among friends. But, after I got married, I came to know about different contraceptive methods from my married friends who had used these methods…I also went to the health post nearby…they explained to me in detail.* - *F16: I heard it in the radio. There was a role-play about family planning.* - *F34: I don’t know where we get these* (Family planning) *services and how to use…* - F36: *I do not use family planning because I do not know where, what, and how to use and I feel shy to ask others* - *F23: We don’t know what methods are available for female. So, we don’t use any.* - *I2: We don’t know about any* (family planning) *measures. Nobody told us about them. Though we did not use it, I now feel that it is an important requirement. It has been very difficult to take care of this large family. I do not want my sons and daughters to go this way. Better to use it* (family planning measures) *by them.* - *I8: People from Madhesi and Muslim community are unaware of family planning services whereas the Brahmins and the Chhetris are using the methods maximum among all.* |
| Perceptions of FP | - *F9: Family planning is a tool for population control. It should be used by every married couple.* - *F4: My sister used to say that she has been using injection* (Depo-Provera) *to control unwanted pregnancy. I think family planning is about the same.* - *F26: After you have a desired number of children, then you can do the operation after which you are unable to conceive. I think family planning is said to be the same thing.* - *F7: It is a process that postpones the birth of a baby. It can be due to the natural periodic or withdrawal method as well as using contraceptives.* - *F10: It is the thing that has to be used by females. So, they might know much more about it.* - *F13: Family planning is the use of condoms during intercourse.* |
| **Preference for FP methods and decision making** | |
| Preference among participants | - *F5: It takes an hour to reach the nearest health facility….who bothers to go there in this scorch. Rather I use the natural method.* - *F4: One of my sisters used Depo injection for it* (family planning)*. Later, she started having continuous bleeding and abdominal pain. It was very difficult for her to cope. So, we are afraid of using any methods.* - *F13: We use any methods that are available in our health center. Easiest one is to use condom, which has no side effects.* - *I1: Family planning has been an issue of females for a long time. Males are reluctant to use those* (FP measures*). Most of our clients who come for it* (family planning) *are females. Even they* (females) *come to take condoms. There are rarely any males or any couples coming for family planning.* |
| Decision-making roles in the family | - *F15: It is an important concern. I will let my wife do it.* - *F7: The case is different in our home. My husband is an alcoholic. He goes to the factory to work the whole day. He is angry with me if I say or decide anything by myself.* - *F20: My husband is the only person in my home who earns money. He has been looking after every stuff at home. So, I believe in him. I respect his decision about whether to use any methods or not.* - *I3: Though I am interested to do, I am weak. I am taking medicines for hypertension. If I opt for it* (sterilization) *myself, then I will get paralyzed. I will lose my immunity too. Who would look after my family afterward?* - *F15: Though I love my wife and I am concerned about her. But I have no options. I must work in a factory. I need to lift heavy weights there. All the major house chores are also done by me. These things (sterilization) would make me weak. How can I earn my livelihood then?* - *I5: Family planning is targeted commonly for females because many of the methods are for them…. but the paradox is the decision-makers regarding their* (contraceptives) *are the males. It’s ok to discuss with their husbands but their (wife) decision should be respected.* - *I2: Female has to go through various phases. They must bear menstruation, must be pregnant, and many others. So, undergoing permanent sterilization by females makes them weaker.* - *I8: It’s not that one person should be the decision-maker. It should be on the mutual understanding of the couple.* - *F6: I think I would be using it* (Contraceptives)*. It is our culture to respect our husbands. How can I force him to use it* (Contraceptives) *by him* (husband)? - *F12: It* (Family planning) *is stuff to be done by the females. So, there is no doubt about who would be doing. Moreover, people would laugh at me if I do.* - *I8: Females have already gone through much pain in bringing up and taking care of the children and again keeping this stuff (family planning) in their head is unjustifiable. As such, in comparison to the female operative procedure, I have heard that male one is simple, less time consuming, and do not bring many complications. So, why not we male take lead on this?* - *F16: I declare that I will not let her* (wife) *use any family planning methods. Rather, I will use them.* |
| **Barriers and challenges on the utilization of family planning** | |
| Supply-side barriers and challenges | - *F4: Going to health centers is not possible for us, as they are very far from here. Thus, it is better not to use them* (contraceptives). - *F1: My husband is the only person in my family who earns money so hard…we have many things in priority so it is difficult to buy them (contraceptives) every time.* - *I14: 2 years ago, I went to the health post to use Implants. But that day, the trained worker was on holiday. I returned home a long way on that hot day. Again, I went next time after 2 months. This time, the health worker was already transferred to another health center and there was no one to provide service. Since then, I never went health post again.* - *F47: They don’t give much importance to us (boys). They only look at pregnant females and married ones. They are themselves not clear that family planning is not only for couples. Better to change the name* (family planning) *as it is not only for the ones who have a family.* - *F23: Everyone is in the same place there* (health center)*. I feel awkward to talk about it (family planning) in front of others, especially males. It would be better if they have separate private rooms for counseling or distributing contraceptives.* - *F22: Our preferred choice of contraceptives is not provided as they are not available. A few months back, it was ok. But now, the supply is low.* - *F43: Detailed chapters regarding reproductive health and family planning should be kept in our curriculum. We get to know many things from our school.* - *F38: Though we are eager to learn about those lessons (reproductive organs and health), our teacher skips them. They tell us to read it by ourselves.* |
| Demand-side barriers and challenges | - F39: *I do not use family planning because I do not know where, what, and how to use and I feel shy to ask others.* - *F40: My husband works abroad. Last year, when he came home during Dashain (festival). We had it (intercourse). Later, he returned to his workplace. Meanwhile, I came to know that I was pregnant, after 3 months. I was shocked to hear that. We already had 4 children; 2 of them were unplanned. I did not have enough information about contraceptive measures in this situation. Had I known about them; I would have used. I had serious trouble traveling to get it aborted.* - *F35: After the birth of my second baby, I started to use injection* (Depo-Provera)…*it didn’t last longer…I experienced bleeding and abdominal pain…. so left it* (Depo-Provera) *and started taking pills.* - *F18: I used implants for a long time, but it disturbed my menstruation cycle…. sometimes it occurs in 15 days, sometimes it even takes 4-5 months ….so I left using.* - *F24: I have a much bitter experience. I was using Depo injection before. But I started having over bleeding for which I was admitted to the hospital for a few days. Later, I was switched to implants but they also did not suit me. In between I also used pills, but they aggravated my acne and I was feeling nauseated every day. Uff…. I am fed up now. I swear, I won't ever use any methods.* - *F17: I have heard that keeping these things (Copper-T) in the uterus can cause cancer. Better to avoid it.* - *I18: There aren’t any choices for males. I think using a condom during sex is like tying plastic around the tongue and eating food.* - *F21: We don’t get any ‘use and throw’ methods for females like male condoms. Otherwise, we would use it when our husbands deny using condoms.* - *F3: There’s a belief in our community that the use of birth controlling pills mean disrespecting our gods.* - *F37: Though family planning is not openly talked about in the society, when the couples plan not to have babies for 3-4 years, the relatives and other close family members scold them or tease them of not capable of giving birth to children.* - *I12: Some husbands working abroad forbid their wives from using any FP measures because they fear the use of family planning measures may provoke the sexual relationship with someone else in their absence.* - *F19: My husband and I decided to postpone our pregnancy for a few years. I kept implants over my arms. It had been 3 years since we married, and people around us started back-biting me. They blamed me that I was infertile and stopped speaking with me. My husband was even told by my father- and mother-in-law told my husband to marry another girl. Then I removed the implants and prepared myself for pregnancy though I was mentally not ready. Now, I have 3 children. I had to leave my study in the middle due to family pressure.* - *F20: My aunt gave birth to a son after 5 successive daughters. She is pregnant again this time in the hope to have a son. She says that she cannot trust to have only one son because if anything happens to their only son, then she will have no one to pay tribute after her death.* - *I10: How can we tell people to use family planning? It is a sin to talk openly regarding it* (family planning)*. We never used these* (contraceptives) *in our lifetime, yet we are happy. Children are the gifts of god. Instead, it is a moment of pride. So, there is no point in stopping birth. It is against the law of nature, and our religion and culture.* - *F26: Though nobody speaks up only on a matter of family planning, they are using the methods privately.* |
| **Role of youth and suggestions to improve family planning** | |
| Youth’s engagement on strengthening family planning services | - *I16: Mobile clinics should be started immediately…. outreach activities need to be extended in our village too. It will obviously help reach these (contraceptive) services to a greater number of people.* - *F24: We don’t know of any youth-led activities in our place. Sometimes a few people from outside come to our place, but they are either adults or old people. You people* (to the research team) *are the first we have seen in our place from outside our village talking about family planning. We are happy to see you here.* - *F42: Since the youth are the active members of the society, they can disseminate FP information to other people as well.* - *F35: We can help people in using FP measures by organizing awareness programs in the community and collaborating with some youth clubs here. But beforehand, we should be given adequate training.* - *F17: Moreover, our teachers, doctors, politicians, leaders, priests should be made advisors…they are influential here (community)….elder people will not agree with what we say….in addition, it’s awkward for us to talk in this issue with our elders.* - *F13: I feel bad for my sister who is not given much importance from my parents. She got married against her choice due to parents’ pressure. Now, they are forcing her to have kids. She is just 15 and if she gets pregnant, what will happen to her health and her child, how can she take care of a baby? I had a long debate with my father yesterday. I have now decided to start a youth club to promote awareness regarding FP and preventing early marriage and teenage pregnancies.* |
| Suggestions for improvement of family planning services | - *I25: People should be assured that they* (contraceptives) *do not cause any reactions and side-effects. New methods should be brought. Moreover, more alternatives should be brought for the males, to increase compliance and decrease the burden to females.* - *I6: I admit, most of the couples don’t have a proper talk between them regarding family planning. Spousal communication is also an important aspect of increasing family planning uptake. We males will take lead on it.* - *F20: For a long period of time, females have been using those (Contraceptives) by hiding. We are always in fear about what others would say if they came to know about us using it. This can be addressed through male involvement and support.* |
